# Research Electronic Data Capture (REDCap) - planning, collecting and managing data for clinical and translational research

**DOI:** 10.1186/1471-2105-13-S12-A15

**Published:** 2012-07-31

**Authors:** Paul A Harris

**Affiliations:** 1 Department of Biomedical Informatics, Vanderbilt University, Nashville, TN 37208, USA

## Background

REDCap (Research Electronic Data Capture) is a software application and workflow methodology designed to collect and manage data for research studies. REDCap study databases are secure, web-based applications and easy to create, launch and manage on a project-by-project basis. REDCap uses a study-specific data dictionary to eliminate all programming requirements for the creation of electronic case report forms and participant survey instruments for individual studies – making it extremely fast to develop and launch for any size study. Vanderbilt developed and launched REDCap in 2004 and began sharing the software with other academic and non-profit institutions in 2005 at no cost under a unique consortium dissemination model. The consortium now consists of 322 academic and non-profit partner institutions across six continents serving 38,600 end-users (http://www.project-redcap.org). This presentation will provide a description of the REDCap software platform, global consortium and low-cost institutional models for supporting data management across the entire clinical and translational research enterprise.

